# 1702. Disseminated Candidiasis after Candidemia: Epidemiology, Diagnostic Evaluation, and Risk Factors

**DOI:** 10.1093/ofid/ofad500.1535

**Published:** 2023-11-27

**Authors:** Daniel Whitehurst, Catherine R Murphy, Zheyi Teoh, Caitlin N Brammer, Kerrigan Perkins, Grant C Paulsen, Lara A Danziger-Isakov, Hilary Miller-Handley, William R Otto

**Affiliations:** Cincinnati Children's Hospital Medical Center, Cincinnati, OH; Cincinnati Children's Hospital Medical Center, Cincinnati, OH; Cincinnati Children's Hospital Medical Center, Cincinnati, OH; Cincinnati Children's Hospital Medical Center, Cincinnati, OH; Cincinnati Children's Hospital Medical Center, Cincinnati, OH; Cincinnati Children's Hospital Medical Center, Cincinnati, OH; Cincinnati Children's Hospital, Cincinnati, Ohio; Cincinnati Children's Hospital Medical Center, Cincinnati, OH; Cincinnati Children's Hospital Medical Center, Cincinnati, OH

## Abstract

**Background:**

In patients with candidemia, evaluation for disseminated disease with ophthalmological exam and diagnostic imaging is recommended but not standardized. This study sought to describe practice patterns in the diagnostic evaluation for disseminated disease after candidemia and identify risk factors associated with disseminated candidiasis to improve local management.

**Methods:**

A retrospective study of all episodes of candidemia diagnosed in patients < 25 years from 2013-2022 at Cincinnati Children’s Hospital Medical Center reported clinical characteristics, microbiology data, and diagnostic testing for dissemination. Disseminated candidiasis was defined as an ophthalmologic exam, echocardiogram or other imaging findings consistent with candidiasis or growth of *Candida spp.* from a sterile site besides blood. Mixed-effects logistic regression was performed to identify risk factors for disseminated disease.

**Results:**

144 episodes of candidemia occurred in 124 patients , with C. albicans (35%) the most commonly identified species (Figure 1). 107/144 (74.3%) episodes underwent evaluation for disseminated candidiasis, with dissemination in 27/107 (25.2%). Of patients with only 1 positive culture, 5/34 (14.7%) had disseminated candidiasis compared to 22/73 (30.1%) with more than 1 positive culture (Figure 2). Ophthalmologic exams and echocardiograms were done in < 70% of workups (Figure 3). History of prematurity (aOR 17.5, 95%CI 3.1-100.4) and solid organ transplantation (aOR 5.3, 95%CI 1.1-24.6) were associated with disseminated candidiasis when controlling for age, sex, underlying disease, and initial antifungal treatment. Each additional day of candidemia significantly increased the odds of dissemination (aOR 1.5, 95%CI 1.2-1.9).

**Figure:**
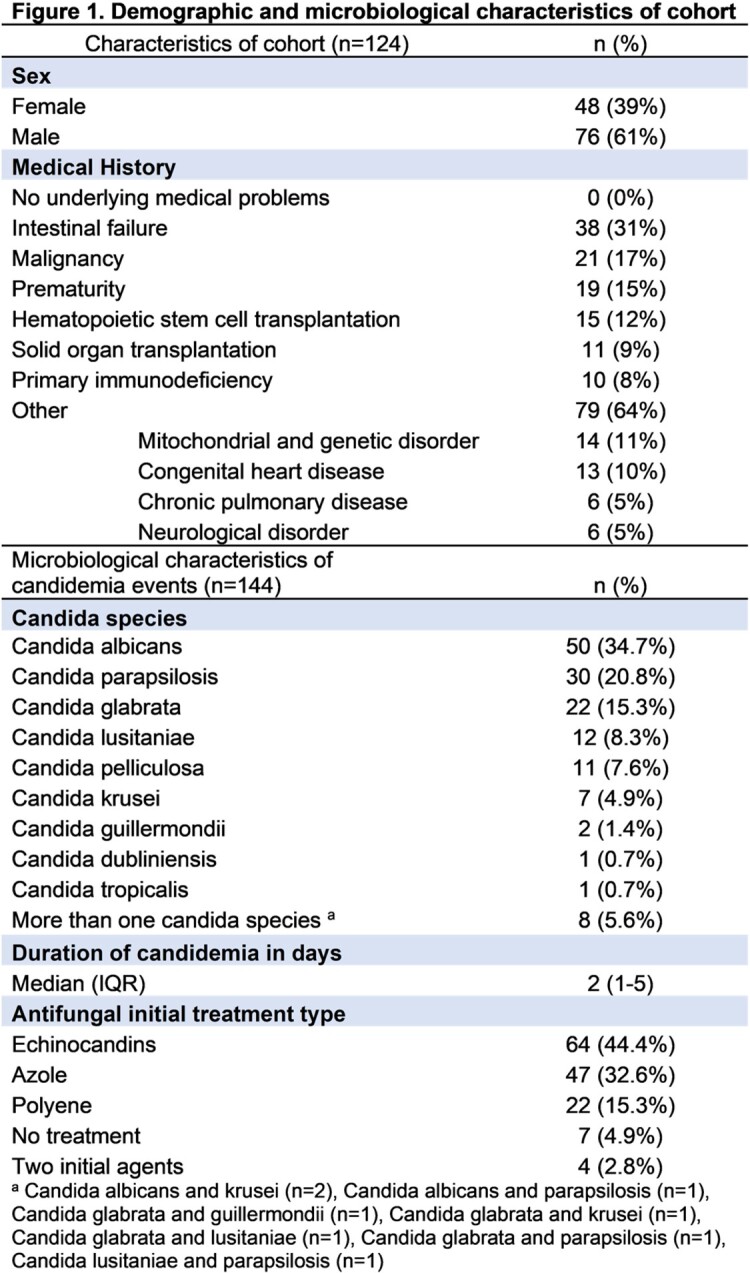

**Figure:**
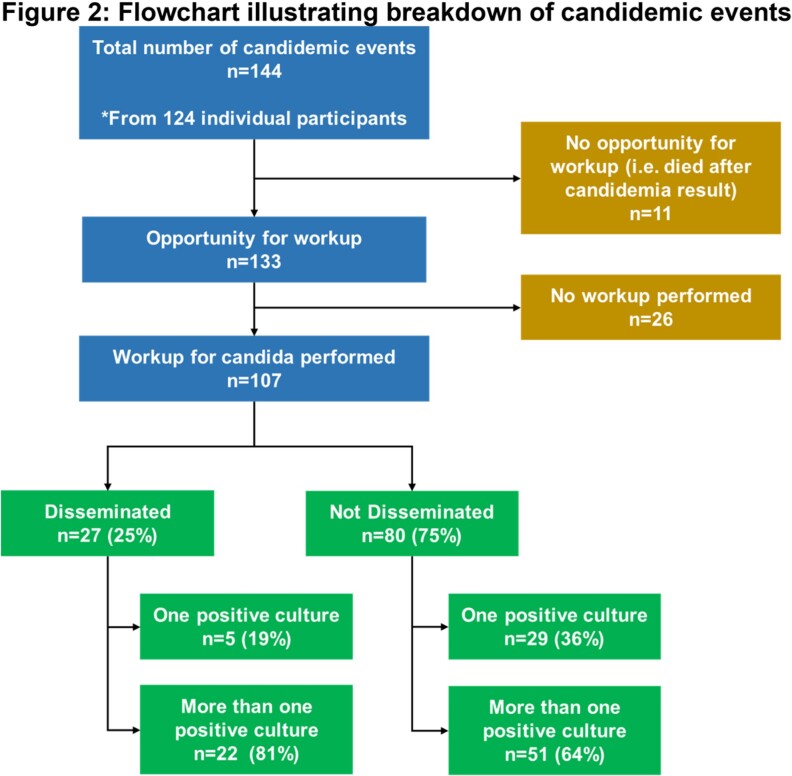

**Figure:**
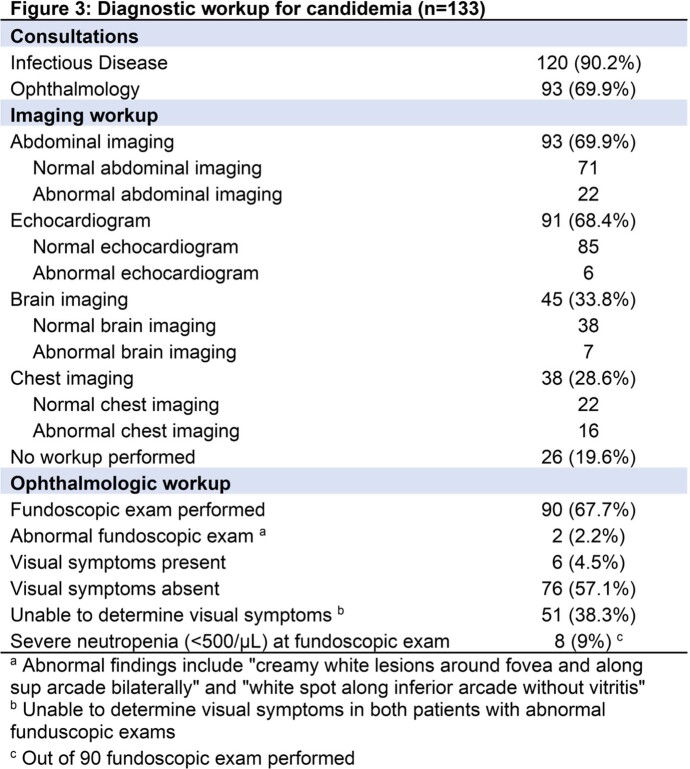

**Conclusion:**

In this single-center study, most patients with candidemia underwent diagnostic evaluation, though the extent varied. Disseminated disease was common. Prematurity and history of solid organ transplantation were independent risk factors for dissemination as was each additional day of candidemia. Patients with any candidemia should undergo evaluation for dissemination. Multicenter studies are needed to determine the optimal diagnostic evaluation in these high-risk patient groups.

**Disclosures:**

**Grant C. Paulsen, MD**, Moderna: Grant/Research Support|Pfizer: Grant/Research Support **Lara A. Danziger-Isakov, MD, MPH**, Aicuris: Contracted Clinical Research|Ansun Biopharma: Contracted Clinical Research|Astellas: Contracted Clinical Research|GSK: Advisor/Consultant|Merck: Advisor/Consultant|Merck: Contracted Clinical Research|Pfizer: Contracted Clinical Research|Roche Diagnostics: Advisor/Consultant|Takeda: Advisor/Consultant|Takeda: Contracted Clinical Research **William R. Otto, MD, MSCE**, Moderna: Grant/Research Support

